# Supplementary figures and images for: Automatic time in bed detection from hip-worn accelerometers for large epidemiological studies: The Tromsø Study
Source: PLoS One. 2025 May 6;20(5):e0321558. doi: 10.1371/journal.pone.0321558 (PMC12054856; doi:10.1371/journal.pone.0321558)

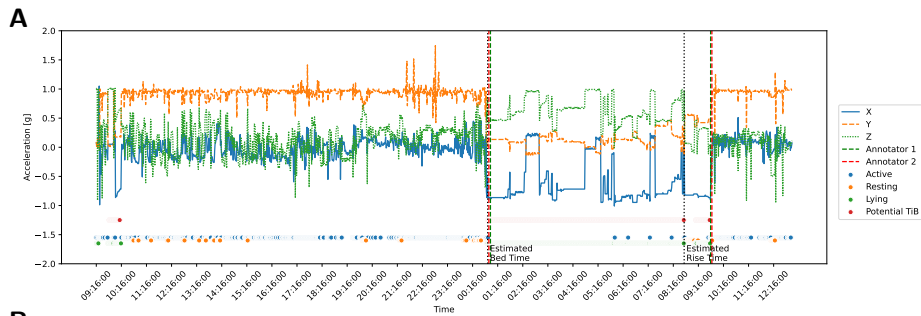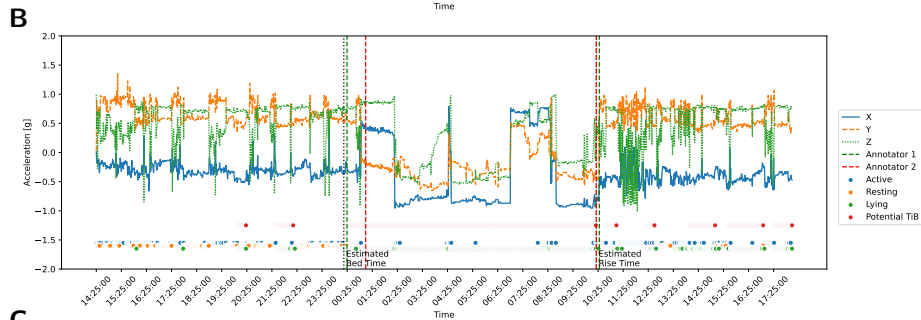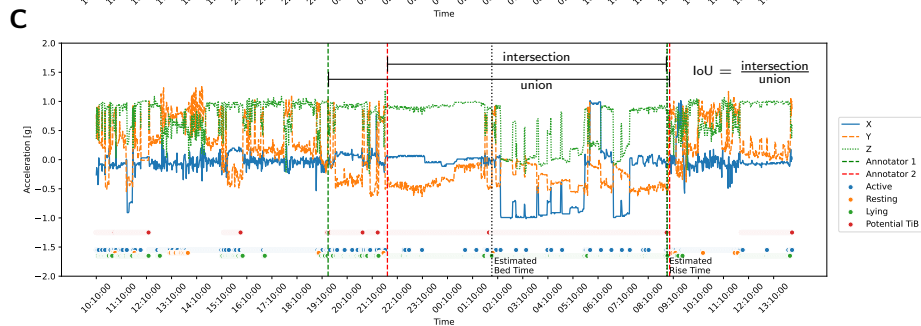

Supplement: S1 Fig — Green and red dotted lines represent the annotations of the two annotators. All other information were visible to the annotators during annotation. (A) Both annotators labeled bed and rise time very similarly (high IoU score) and jointly overruled the suggested rise time (dotted line). (B) Both annotators labeled rise time very similar, but had some disagreement on the bed time (lower IoU score than (A), but still high). While annotator 1 agreed with the suggested bed time, annotator 2 labeled a later onset. (C) Both annotators agreed on the suggested rise time, but labeled the bed time very differently (low IoU score). IoU scores were calculated for all annotations by dividing the intersection (time labeled as in bed by at least one of the annotators) by the union (time labeled as in bed by both annotators) (PDF) [file pone.0321558.s001.pdf]

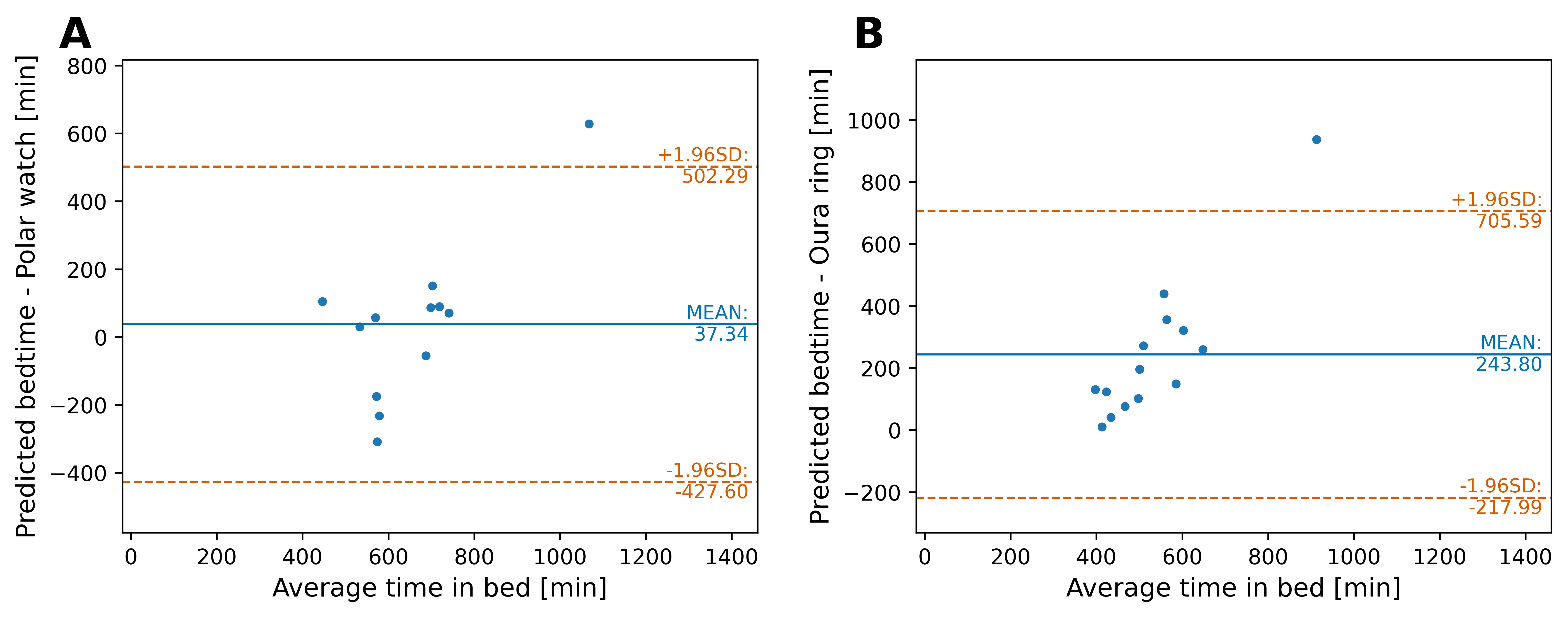

Supplement: S3 Fig — (A) No systematic distortion is visible for the Polar Vantage. (B) The estimates of our model seem to overestimate the Oura ring’s total sleep duration the longer the sleep/TiB is. (PNG) [file pone.0321558.s003.png]

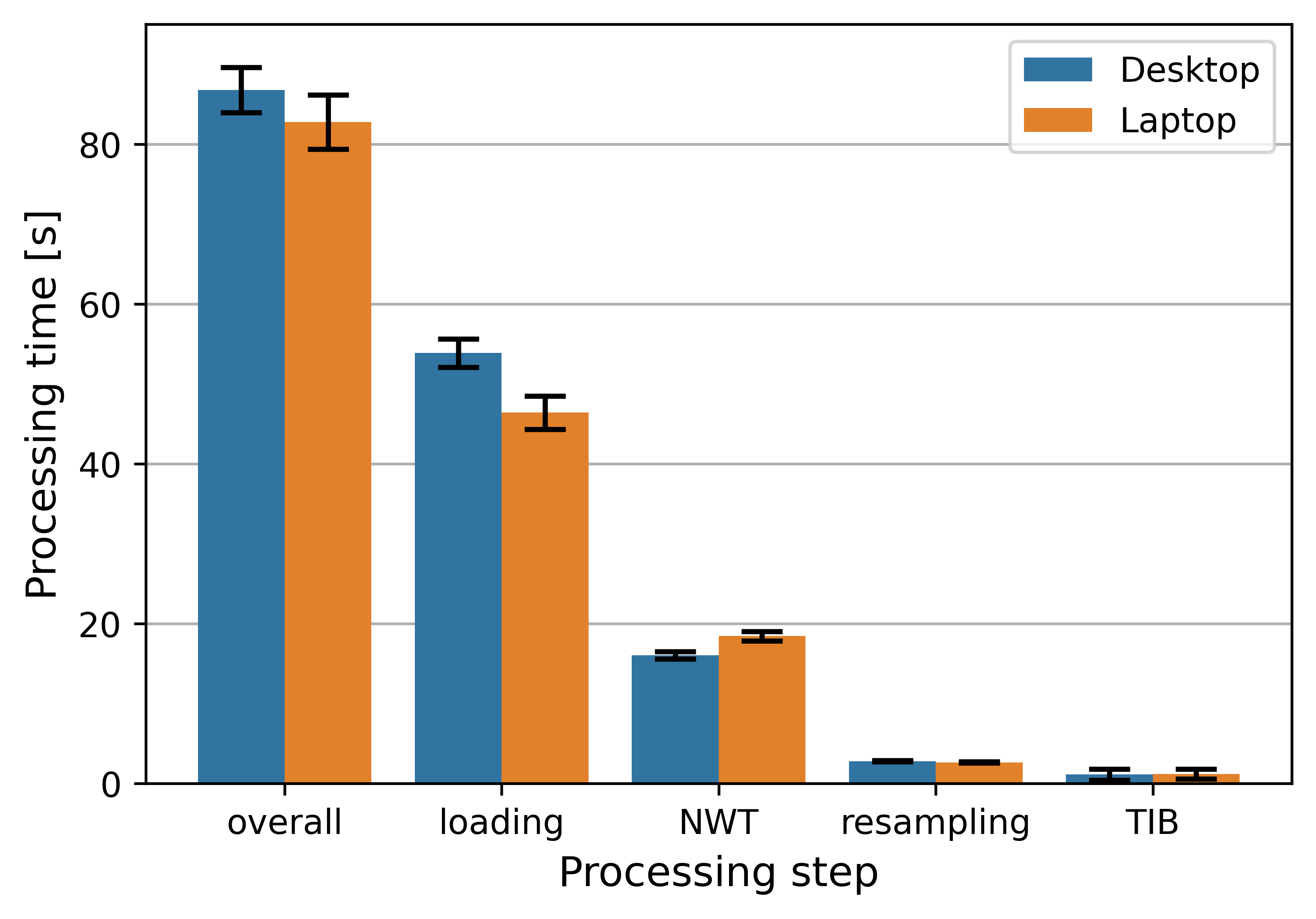

Supplement: S4 Fig — Application of the TiB algorithm is very quick even if the resampling of the acceleration data is taken into account. Reading the GT3X file is usually the slowest step. Processing times were measured on a desktop PC (Intel Core i9-10850K processor with a 64-bit instruction set, 128GB RAM and an Asus GeForce RTX3090 GPU) and a laptop (Intel Core i7-1165G7 processor with 32GB RAM and a 64-bit instruction set). (PNG) [file pone.0321558.s004.png]
