# Supplementary material for: Automatic time in bed detection from hip-worn accelerometers for large epidemiological studies: The Tromsø Study
Source: PLoS One. 2025 May 6;20(5):e0321558. doi: 10.1371/journal.pone.0321558 (PMC12054856; doi:10.1371/journal.pone.0321558)
Supplement: S2 Table — The models reached accuracies up to 94% for unidirectional and up to 95% for bidirectional LSTMs with generally low standard errors across the 10 iterations of cross-validation. Based on the obtained results, we selected a bidirectional LSTM with 4 layers and 32 cells per layer for the further evaluation. (PDF) [file pone.0321558.s002.pdf]

Model	Layers	LSTM cells							
		1	2	4	8	16	32	64	128
Uni-LSTM	1	.90 ± .00	.92 ± .01	.91 ± .01	.92 ± .01	.92 ± .01	.92 ± .01	.92 ± .01	.92 ± .01
Uni-LSTM	2	.90 ± .01	.89 ± .01	.91 ± .01	.92 ± .01	.92 ± .01	.92 ± .01	.92 ± .00	.92 ± .00
Uni-LSTM	4	.79 ± .05	.89 ± .01	.92 ± .01	.92 ± .01	.92 ± .00	.93 ± .00	.94 ± .00	.92 ± .01
Bi-LSTM	1	.91 ± .01	.91 ± .01	.92 ± .01	.94 ± .01	.94 ± .01	.94 ± .01	.94 ± .01	.94 ± .01
Bi-LSTM	2	.91 ± .01	.92 ± .01	.94 ± .01	.94 ± .01	.94 ± .01	.95 ± .01	.95 ± .01	.95 ± .01
Bi-LSTM	4	.92 ± .01	.93 ± .01	.94 ± .01	.95 ± .01	.95 ± .01	.95 ± .00	.95 ± .00	.95 ± .01

Uni-LSTM: Unidirectional LSTM; Bi-LSTM: Bidirectional LSTM
