# Supplementary material for: Automatic time in bed detection from hip-worn accelerometers for large epidemiological studies: The Tromsø Study
Source: PLoS One. 2025 May 6;20(5):e0321558. doi: 10.1371/journal.pone.0321558 (PMC12054856; doi:10.1371/journal.pone.0321558)
Supplement: S5 Table — All metrics were calculated on subject-level using the respective functions of the Scikit-Learn Python package. The table shows between-subject averages and standard deviations (in brackets). (PDF) [file pone.0321558.s005.pdf]

	Accuracy	F1 Score	Recall	Precision	Sensitivity	Specificity	Total TiB	Total predicted TiB
All days	0.920 (0.130)	0.904 (0.141)	0.966 (0.097)	0.875 (0.188)	0.966 (0.097)	0.894 (0.200)	493.471 (93.179)	576.822 (213.178)
w/o outlier predictions	0.933 (0.099)	0.917 (0.115)	0.969 (0.075)	0.890 (0.162)	0.969 (0.075)	0.914 (0.152)	494.305 (91.133)	560.375 (168.907)
w/o outlier predictions and NWT days	0.935 (0.098)	0.917 (0.114)	0.969 (0.076)	0.892 (0.161)	0.969 (0.076)	0.916 (0.150)	491.607 (94.666)	555.291 (169.046)
