# Supplementary material for: Automatic time in bed detection from hip-worn accelerometers for large epidemiological studies: The Tromsø Study
Source: PLoS One. 2025 May 6;20(5):e0321558. doi: 10.1371/journal.pone.0321558 (PMC12054856; doi:10.1371/journal.pone.0321558)
Supplement: S6 Table — 1st, 10th, 25th, 50th, 75th, 90th and 99th quantiles are shown for the three criteria shown in Fig 2, respectively. (PDF) [file pone.0321558.s006.pdf]

	All days		w/o outlier predictions		w/o outlier predictions and NWT days	
	Labeled	Predicted	Labeled	Predicted	Labeled	Predicted
0.01	287.60	255.44	321.04	279.20	275.71	273.04
0.10	392.00	392.00	392.20	392.60	390.00	388.00
0.25	435.00	457.00	435.00	457.00	431.00	449.00
0.50	490.00	530.00	489.00	529.00	488.00	523.00
0.75	543.00	623.00	543.00	619.00	541.00	613.00
0.90	604.00	807.00	601.00	764.20	605.36	758.60
0.99	721.72	1440.00	721.96	1150.12	726.88	1150.12
