# Supplementary material for: Automatic time in bed detection from hip-worn accelerometers for large epidemiological studies: The Tromsø Study
Source: PLoS One. 2025 May 6;20(5):e0321558. doi: 10.1371/journal.pone.0321558 (PMC12054856; doi:10.1371/journal.pone.0321558)
Supplement: Attachment — Submitted filename: response_to_reviewers.pdf [file pone.0321558.s007.pdf]

Response to Editor and Reviewers

Dear editor,

dear reviewers,

We thank you for your constructive and valuable feedback. We hope we have addressed your concerns to your satisfaction. Please find below our responses to each of the points raised.

Best regards

Marc Weitz

Response to the academic editor

1. Please ensure that your manuscript meets PLOS ONE's style requirements, including those for file naming. The PLOS ONE style templates can be found at

https://journals.plos.org/plosone/s/file?id=wjVg/PLOSOne_formatting_sample_main_body.pdf and

https://journals.plos.org/plosone/s/file?id=ba62/PLOSOne_formatting_sample_title_authors_affiliations.pdf.

We carefully read style templates and adjusted our manuscript accordingly. We corrected the corresponding author marking and mail address formatting. Please let us know if you find any other style violations.

2. We note that you have indicated that there are restrictions to data sharing for this study. PLOS only allows data to be available upon request if there are legal or ethical restrictions on sharing data publicly. For more information on unacceptable data access restrictions, please see http://journals.plos.org/plosone/s/data-availability#loc-unacceptable-data-access-restrictions.

Before we proceed with your manuscript, please address the following prompts:

a) If there are ethical or legal restrictions on sharing a de-identified data set, please explain them in detail (e.g., data contain potentially identifying or sensitive patient information, data are owned by a third-party organization, etc.) and who has imposed them (e.g., a Research Ethics Committee or Institutional Review Board, etc.). Please also provide contact information for a data access committee, ethics committee, or other institutional body to which data requests may be sent.

There are in total three data sets used in this study: The Tromsø Study TiB dataset, the Tromsø Study accelerometer dataset, and the ActiGraph/Oura/Polar validation dataset. All these data sets are secondary data that have been and can be obtained from the respective owners. We reached out to the respective data owners regarding your request of openness of the data. Please see the respective responses below.

For the Tromsø Study TiB dataset and the Tromsø Study accelerometer dataset, the legal restriction on data availability is set by the Tromsø Study Data and Publication Committee to control for data sharing, including publication of datasets with the potential of reverse identification of de-identified sensitive participant information. The data can, however, be made available from the Tromsø Study upon application to the Tromsø Study Data and Publication Committee. Contact information: The Tromsø Study, Department of Community Medicine, Faculty of Health Sciences, UiT The Arctic University of Norway; e-mail: tromsous@uit.no. We reached out to the Tromsø Study about the possibility to publish the data used in this study, but because of the legal restrictions, we expect this process to take time.

The ActiGraph/Oura/Polar validation dataset has been collected by Henriksen et al. (2022). The authors plan to publish the data openly, however, currently it is only available upon request to the study's corresponding author. While there is an overlap between the authors of this study and Henriksen et al. (2022), we do not own this data and have therefore no possibility to make it openly available.

b) If there are no restrictions, please upload the minimal anonymized data set necessary to replicate your study findings to a stable, public repository and provide us with the relevant URLs, DOIs, or accession numbers. For a list of recommended repositories, please see

https://journals.plos.org/plosone/s/recommended-repositories. You also have the option of uploading the data as Supporting Information files, but we would recommend depositing data directly to a data repository if possible.

We will update your Data Availability statement on your behalf to reflect the information you provide.

Please see the information provided under 2a, why we cannot share the data.

3. In the online submission form, you indicated that [The data of the Tromsø Study cannot be shared publicly because of the risk of potential reidentification, but can be obtained upon application to the Data and Publication Committee for the Tromsø Study: https://uit.no/research/tromsostudy. The ActiGraph/Oura/Polar validation dataset can be obtained from A.He. (andre.henriksen@uit.no) upon reasonable request.].

All PLOS journals now require all data underlying the findings described in their manuscript to be freely available to other researchers, either 1. In a public repository, 2. Within the manuscript itself, or 3. Uploaded as supplementary information.

This policy applies to all data except where public deposition would breach compliance with the protocol approved by your research ethics board. If your data cannot be made publicly available for ethical or legal reasons (e.g., public availability would compromise patient privacy), please explain your reasons on resubmission and your exemption request will be escalated for approval.

Please see our statement under 2a, why we cannot share the data.

Response to the reviewer #1:

Reviewer #1: This paper presents an algorithm aiming at discriminating time in bed and time out of bed based on hip-worn accelerometry data. While hip-worn accelerometry data focused on physical activity, such an algorithm would enable to reuse this data to get other outputs related to sleep. This algorithm is based on an augmentation data process and machine learning models of type Long-Short Term Memory networks (LSTM). This study has potential because the procedure used well-known algorithms applied in similar contexts and some techniques to add noise or take into account potential misuse of devices (flipped sequence, Gaussian noise), but there are weaknesses that need to be revised before publication.

General comments

As you mentioned in the paper, wearing hip-worn accelerometers during 24h can be a burden for participants, especially during sleep hours ("wrist-placement has been considered less obtrusive and thus leads to fewer non-wear days"; "To decrease study participant burden, many early study protocols comprised the removal of the device during sleep"). This fact might explain the lack of data appropriate for this kind of study. I think you should discuss the potential of using hip-worn accelerometers in behavior monitoring, maybe in future studies. In addition, it is probably a limit to the study, not so much a scientific limit as a functional one. It should be mentioned.

We are now discussing the potential in the first paragraph of the discussion and mention explicitly two of the larger studies that currently use hip-worn accelerometers with a 24h wear protocol: The Tromsø Study and the Nako Study. The benefits of hip-worn accelerometers with respect to physical activity alone are discussed in the introduction.

In your process, you use many types of data but it is unclear from the description how this data is used in the procedure. For instance in the procedure, it is not clear which dataset was used to train the models. Is it the "Tromsø Study TiB dataset", "the Tromsø Study

accelerometer dataset" or a mix of both? Please clarify the function of each dataset you used.

We have split the Procedure section into three subsections (Data preparation, Model training, and Model evaluation) and clarified in each subsection which dataset was used for which step and purpose. We further included an overview figure in Figure 1.

I am a bit skeptical about the use of ActiGraph/Oura/Polar dataset. Only 15 (16 – 1) participants provide "relevant information". Moreover, it seems there is a confusion between sleep duration and time in bed (please consider my comments / questions related to the procedure section).

We fully understand the skepticism about this dataset and strongly acknowledge its limitations. However, we believe that, despite its limitations, this dataset addresses an important issue of this paper, that is the generalization of the model. To avoid the impression of overinterpreting this data, we extended the discussion of its limitations, and also stated the objective of its use more clearly. We also agree on the difficulty of comparing TST with TiB, but again, our objective with this dataset was to test for "reasonable results" in the sense of slightly higher but not far off TiB estimates compared to TST. We changed the text to make this point clear.

You mentioned that "data augmentation" improved the model generalization. How this technique improved the model. There is no comparisons with and without data augmentation. I don't understand how you can make such a claim. Additional information is needed, please consider my comments / questions related to the discussion section.

We rephrased this claim as we did not find a good way of quantifying this as the matter is more complex than only accuracy comparisons. We also highlighted now that our choices here were heuristically guided and motivated by data augmentation in other disciplines such as computer vision and natural language processing. Our intention was not to focus this paper on this topic (even though we believe that this is a crucial topic currently missing in the literature) and that we would have liked to make more informed decisions about augmentation than the heuristically motivated choices we have made. In fact, our

intention was to point out this knowledge gap in the discussion, and we hope that we have made this clearer now. Finally, we want to point out that we are doubtful that the question of augmentation can be solved empirically in singular experiments. We are at least not aware of any papers in the above-mentioned disciplines who did that. Our impression from these disciplines is that over time some consensus/ideas of which augmentation is reasonable on which task has emerged. So, we hope that in the discussion, we managed to make clear now that regarding the augmentation we are using here, we neither claim to be complete nor correct, but that it is reasonable to apply it.

Abstract

The method description should be improved in the abstract. For instance, you wrote “We introduced new and accelerometer specific data augmentation methods”: could you briefly describe these new methods?

Given the limited space in the abstract, we could only include a very brief explanation of the augmentation methods used. For the more interested reader, a longer description is provided in the methods section.

“the trained model significantly over- or underestimated time in bed.”: could you add numeric indicators showing this over or underestimation?

Our intention with this sentence was to refer to the few cases in which the model predicted all or nothing as time in bed (please see in Fig 1B the points at 0 and 1440 minutes of predicted time in bed, which we marked as "outlier predictions"). We added this information now in the respective sentence and replaced significantly by fundamentally to avoid confusion with statistical significance.

Materials and methods

“To ensure data quality, only participants with an intersection over the union (IoU) score of over .9 between the two annotations were included in this study.” (l 91): Please clarify this sentence. For instance, what are “the annotations”? Are they the annotators’ labeling? How was computed the score?

We now consistently use the term "annotators' ratings" (as used already a couple of times in the text) to avoid confusion. Please indicate if this term seems misleading to you. We also added a description of how the IoU score was computed. A visualization and the mathematical formula are now included in S1 Fig.

“Each participant was maximally included once in this subset.” (l 101): What was the criterion for choosing one of the 5 days (7 – 2 days) for each participant?

We are not 100% sure about the exact meaning of this question but anticipate that our description of the sampling procedure is confusing. For that reason, we added more information about the accelerometer data in the Tromsø Study (~6000 participants with 7 days recording each) and that we sampled 500 days from these ~42000 days to have a feasible sample of approximately the same size as the TiB dataset providing additional heterogeneity (e.g., people with potentially different sleeping behavior).

Procedure

It seems that you use two different methods to label time in bed minutes. On one hand, you use Actiwave Cardio data from the “Tromsø Study TiB dataset” to label the TiB minutes from the ActiGraph sensor data. On the other hand, you use ActiGraph data from the “Tromsø Study accelerometer dataset” to label the TiB minutes from the ActiGraph sensor data.

• Why don't you use the same method to label the ActiGraph sensor data? It should be explained. It is important to show that no bias could result from the use of two different methods, or at least that this provide a gain in your study.

• What is the discrepancy between the two methods? This could be an important information to add in supplementary materials if you explain and highlight the importance in using these two different methods.

The method for the TiB and the accelerometer dataset is largely the same except that for the TiB dataset, we had an additional sensor (the ActiWave Cardio) available which we utilized for this dataset in addition. The ActiWave Cardio provided additional and reliable inclination data as it was glued to the participants' chest. This additional inclination data is also visualized now in supplementary material S1. For the accelerometer dataset only the ActiGraph was available. We now clarified this in the respective dataset descriptions. We also added a brief statement about our motivation for the Tromsø Study accelerometer dataset and highlighted the similarities (same overall labeling procedure) and differences (Availability of the ActiWave Cardio data only in the TiB dataset) to avoid confusion.

"Also with 50% likelihood, the sequence got reversed." (l 126): Why did you reverse the sequence? What do you expect to simulate with this?

We added our intention that this can simulate inverse turning behavior (e.g. adding a left-to-right turn in the night from a right-to-left turn)

"As for the ActiGraph/Oura/Polar validation dataset only total sleep duration data was available, we calculated the total TiB from the predictions of our model on all fully recorded days." (l 137): If I well understand, this means that you compared the sleep duration from ActiGraph/Oura/Polar with the time in bed from your model predictions, but these features (i.e. sleep duration and time in bed) are generally different. I think this is an important bias when using the ActiGraph/Oura/Polar validation dataset. This may explain the highlighted discrepancy in your results. Have you considered deriving total sleep time to time in bed or the reverse before proceeding the comparison?

We acknowledge that these are fundamentally different measures, and it is very hard/impossible to compare them. However, please note that as we have pointed out now

more clearly in the text, the motivation to include this dataset was to primarily obtain a better idea on whether the model's predictions are reasonable on demographically different data than the data it was trained on. This information could not be gained by the TiB dataset as it, even though its different data than in the training, shares comparable demographic characteristics. We also want to note, that this data is, without any doubt, not the most ideal data and has many shortcomings (the sample size, much more NWT than for instance the Tromsø data) and we would have loved to validate our model on "better" data, in the sense of more fitting labels. However, such data is not available to our knowledge. Nevertheless, we believe that also given all these shortcomings, the ActiGraph/Oura/Polar validation dataset provides an indicator that the model does at least not completely fail on demographically different data.

We now discuss the motivation and limitations of this dataset in more detail and express the above-mentioned line of thinking also in the text.

Adding a flow figure showing the procedure and how the different datasets were used would be helpful for the reader.

We added an overview chart containing key features of the three datasets and how they were used in Figure 1.

Results

You provided the accuracy in your results, but what about the sensitivity and specificity?

We examined sensitivity and specificity in the process but omitted it as we did not have the feeling to gain much more information but acknowledge the interest in these metrics. We have now included sensitivity and specificity in the results section and in more detail together with other (more machine learning related) metrics such as F1 score, recall and precision in the supplementary material S5 Table.

Similarly, you provided the accuracy related to the “Tromsø Study TiB dataset” and the “Tromsø Study accelerometer dataset”, and what about the TiB labeled, TiB detected,

etc.? Additional indicators (average of minima, maxima or means, etc. for instance; you could consider root mean square errors as well...) used to compare the output and the expected results would be relevant.

Minima, maxima and distributional information can be obtained from Fig. 1B. We did not explicitly state these as some "outlier predictions" caused them to be 0 and 1440 min (24h), respectively, and thus the stated minima and maxima would highly depend on the choice of outlier criterium. However, with Fig. 1B, we hope that the interested reader can infer this information based on their own outlier definition. However, following your thought we included a quantile table in the supplementary material S6 Table with the 1st, 10th, 25th, 50th, 75th, 90th and 99th quantile for the three criteria shown in Figure 1, respectively.

Discussion

Please, consider these two sentences "It remains also an open question to which extent our results generalize to the detection of sleep, which we could not test within the scope of this study as no appropriate data from hip-worn accelerometers is currently available." (l 181) and "A crucial strength of this paper is to provide an opportunity to estimate TiB from already collected data. With this, we anticipate the chance for longitudinal analyses of sleep and TiB-related research question in settings where these questions only recently got into focus, but prior data exists." (l 219) I am a bit confused. There seems to be a contradiction between these two statements about the availability of data on which to apply your method. Could you clarify what you mean?

We do not believe that there is a contradiction here but see that these sentences are confusing. We changed the first sentence to clarify that here we mean "with sleep times annotated acceleration data from the hip" which should ideally be openly available (to validate models and algorithms). By this, we hope to clarify what data is meant here compared to the existing (only) hip acceleration data (e.g. in the Tromsø Study, the German National Cohort Study, etc.).

“Another important contribution of this study is the introduction of accelerometer-specific data augmentation as a tool to improve model generalization.” (l 230): I am not sure that the reader well understands the point of using this technique in the procedure. As this is not the purpose of the article, could you please add a table in supplementary materials showing the quantified gain in accuracy between model basically trained and a model trained with data augmentation?

We expanded the discussion of the data augmentation now and added our motivation which is based on the consensus found in other areas where Machine Learning has become dominant like computer vision or natural language processing. We also believe that measuring the effects of singular or combined augmentation methods is not simple as this cannot only be described in terms of accuracy. For instance, we initially struggled more with different predictions for the same data if the prediction onset was set to different time points (e.g. if you would remove some time in the beginning of your data, you would get different predictions). For that reason, we clearly stated now that this study does not quantify the data augmentation effects (which is certainly a whole task) and we are not familiar with similar papers in computer vision. We think this is likely because it is very difficult to quantify and additionally extremely task specific. We mainly want to inform other researchers here to consider using data augmentation for their tasks and hope that as with other disciplines, some consensus is formed over time which augmentation methods are suitable for which tasks.

Figure S1

It would be helpful in Figure S1 to show which participant met the requirements fixed in your study, and for those retained, to describe (for instance, with another color and a thick, continuous line) which parts were considered as time in bed according to your criteria.

The three participants shown in S1 were selected to show different annotation results (high agreement, mediocre agreement and low agreement, respectively), but we noticed that we did not clarify this sufficiently. We aimed to add as little additional information in the plot as possible to maintain the impression that the annotators had during annotation (we only added the annotators labels as otherwise the agreement information could not be preserved). We now extended the caption of S1 Fig. and added a visualization of the IoU score in subfigure C.

Figure S4

What are the characteristics of the machines: desktop and laptop (processor, RAM, 64 or 32 bits)?

We added CPU, RAM and instruction set for both PCs and GPU information for the desktop to the caption of S4 Fig.

Typos

Introduction (l 21): descriptopn -> description

Results (l 148, 152): hyperparamter -> hyperparameter

Discussion (l 207): over -> offer?

Discussion (l 246): a algorithm -> an algorithm

We corrected the mentioned typos.

Best of luck for your paper submission.

Thank you!

Response to reviewer #2:

Reviewer #2: The present study examined an important topic in the literature - the use of accelerometer to evaluate sleep. The development of an algorithm using the information of hip-worn accelerometer data would allow the exploration of a high amount of data that previously used accelerometer in the hip. Despite an important topic, the study has some important caveats that should be addressed. See my following comments that should be addressed:

ABSTRACT

The authors should better provide the rationale of the study - what is the potential advantage of measuring time in bed using hip-worn accelerometer? The objective of the study is not clear. The authors should provide more detailed results in the abstract.

We clarified the objective of this study in the abstract. We also added more details about the cases where the model significantly over- or underestimated TiB.

METHODS - Datasets

The authors should provide a more clear and detailed description of the datasets used in the study. Why the authors choose these three different datasets?

We added a more detailed description, highlighted the similarities and differences between the datasets better and added one sentence clearly stating the motivation for each dataset.

In the Tromso Study TiB dataset, where the accelerometers were placed?

We added that the ActiWave Cardio was attached to the skin with electrodes to the participants chest and that the ActiGraph was worn on an elastic band on the right hip.

Line 79 - Why did the participants in the Tromso Study TiB dataset wear the accelerometers only during 24-hours?

The limitation here was the battery of ActiWave Cardio which runs max one and a half day. The ActiGraph was also worn afterwards (for approximately another 6 days), but the time

when data from both monitors was available was limited by the ActiWave Cardio. We specified this now also in the text.

Is the ActiGraph monitor cited in the Tromso Study accelerometer dataset the same than the used in the Tromso Study TiB dataset? This is not clear in the manuscript.

Both datasets stem from the same collection wave and procedure, but only around 10% of the participants who wore an ActiGraph for seven consecutive days also wore an ActiWave Cardio for one day. This information has been added now. For a comprehensive description see also Hopstock et al. (2022) which is also referred to in the manuscript.

The idea of evaluating the accuracy of information comparing with consumer-wearable data is good. However, the very limited information of this validation dataset (n=16) is a reason of concern.

We now state the motivation behind this dataset more clearly and extended discussing its limitations.

Procedure: It is not clear how the performance of the model was assessed.

We specified that for the hyperparameter search performance was assessed by accuracy. We further specified that all performance measures (metrics) were calculated using the widely adopted standard functions from the Scikit-Learn Python package.

A more detailed description of the Van Hees et al. method is required in the manuscript.

A more detailed description of the Van Hees method has been added to subsection "Non-wear time detection" in the "Procedure" section.

RESULTS

Description is poor, without presenting most of statistical estimates.

We now also report sensitivity and specificity in the results section and added a more comprehensive overview of various metrics in the supplementary material S5 Table.
